# Seed germination ecology of *Ageratum houstonianum*: A major invasive weed in Nepal

**DOI:** 10.1371/journal.pone.0225430

**Published:** 2019-11-21

**Authors:** Anju Lamsal, Mohan P. Devkota, Deepa S. Shrestha, Srijana Joshi, Anil Shrestha

**Affiliations:** 1 Department of Botany, Tribhuvan University, Amrit Campus, Kathmandu, Nepal; 2 National Gene Bank, Nepal Agricultural Research Council, Lalitpur, Nepal; 3 International Centre for Integrated Mountain Development, Lalitpur, Nepal; 4 Department of Viticulture and Enology, California State University, Fresno, California, United States of America; Brigham Young University, UNITED STATES

## Abstract

In recent years, spread of invasive alien plant species (IAPS) has been a major concern in Nepal. One such IAPS is *Ageratum houstonianum*, an Asteraceae, that is a prolific seed producer and difficult-to-control in farmland and various ecological regions causing crop yield and biodiversity losses. However, very little information is available on the germination biology and ecology of this species. Therefore, experiments were conducted to assess the effect of water stress, pH level, and light requirement on seed germination, and the effect of seed burial depth on seedling emergence. Water stress was simulated by polyethylene glycol solutions ranging from 0–5.56 MPa and pH solutions ranging from 4 to 9 were prepared using hydrochloric acid and sodium hydroxide. Germination tests were conducted in petri dishes lined with filter paper and placed in a controlled environment chamber set at 20° C. Light requirement comparisons were made by having petri dishes wrapped with aluminum foil or left unwrapped. Seedling emergence was evaluated by placing seeds at depths ranging from 0 to 20 mm in the soil. Results indicated that this species was moderately drought-tolerant because germination ceased beyond 0.51 MPa. Greater germination occurred at neutral to acidic than at alkaline pH levels. The seeds were positively photoblastic because no germination occurred under dark condition. No seedlings emerged from seeds placed more than 2 mm deep in the soil, indicating that this is a primarily surface germinating species. These findings will help predict future invasions and in development of management strategies for this IAPS.

## Introduction

Invasive species are non-native and can include plants, animals and microbes that have been relocated deliberately or accidentally as a result of economic activities [1]. Invasive alien plant species (IAPS) have attracted extensive attention among ecologists, agronomists, foresters, conservationists, farmers, and entrepreneurs because of their negative ecological impacts and associated economic loss at various scales [2]. For example, such species generally alter ecosystems function by changing species composition, fragmenting natural areas, creating degradation, and by reducing biodiversity [3]. Therefore, IAPS have become the focus of intense research and management worldwide [4].

It has been reported that *Ageratum houstonianum* is one such problematic IAPS in Nepal with widespread distribution in the mid-hills and the plain areas along roadsides, protected national parks, agricultural fields, and fallow lands [5]. The authors further state that the local community have identified it as the worst invasive species in agroecosystems. However, there are no reports, other than distribution, on the ecology of this species in Nepal.

*A*. *houstonianum* belongs to the Asteraceae family and it is native to Central America and Mexico. It was brought to Europe from these regions as an ornamental plant [6]. In recent years, it is reported to have a presence in all continents and pose serious threat to different ecosystems including community forests all over the world [7]. This species is a short-lived (annual or biennial) herbaceous plant growing 20–100 cm tall. Although the primary means of propagation is by seeds, it has been reported that it can also propagate vegetatively by stem portions [8]. An understanding of the biology and ecology of IAPS is essential for the development of management options, and this species is no exception. However, very limited information is available on the germination ecology of this species. In comparison, there is more information available on the germination of another congener *Ageratum* species, *A*. *conyzoides*. For example, a study evaluated the effect of temperature and water stress on seed germination of *A*. *conyzoides* [9]. Similarly, a lot more information is available on the biology and ecology of other Asteraceae family species such as *Artemesia* [10, 11] and *Conyza* [12] because of their ornamental value, invasiveness, or resistance to several herbicides globally.

Information on environmental factors affecting the germination of *A*. *houstonianum* specifically could prove to be very helpful in understanding its ecology and development of management techniques not only in Nepal but globally because of its worldwide presence. Therefore, the objective of this research was to develop information on the germination and emergence of *A*. *houstonianum*. Studies were conducted to determine the effects of varying levels of moisture and pH, and presence and absence of light on seed germination, the depth of seed burial on seedling emergence, and the ability of the seeds to germinate on immediate dispersal from the mother plant.

## Materials and methods

### Seed collection

Seeds of *A*. *houstonianum* for the germination and emergence studies were collected on November 24, 2017 and January 16, 2018 for the first and second round of the study, respectively from the Ranibari Community Forest (27°43'52.81"N, 85°19'8.81"E, 1,303 masl) located in the northwestern part of Kathmandu, Nepal. Mature *A*. *houstonianum* plants growing in open areas with mature and healthy inflorescence at this site were randomly selected for seed collection. Seeds were collected from 20 plants randomly in a 10 m radius area by shaking the inflorescence after inserting them inside polythene bags. The seeds from different plants were mixed to make composite samples. The seeds were then transported to the lab and stored under room temperature (approximately 25°C). Seeds for estimating dormancy requirement were collected on July 4, 2018 and July 22, 2018, for the first and second round of the experiment, respectively from Kirtipur (27°67'29"N, 85°28'32"E, 1,330 masl), Kathmandu, Nepal using similar protocols. A preliminary germination test on the collected samples were done by putting some seeds on moist paper towels, under normal room conditions described above, to test for seed viability. More than 90% of the seeds germinated, thus indicating excellent seed viability.

The authors confirm that no permission was needed to collect the weed seeds, as well as that the studies did not involve endangered or protected species.

### Moisture stress at seed germination studies

Experiments were conducted in summer 2018 in a seed germination chamber (Model 67094A Indosaw Double Chamber Seed Germinator, Osaw Industrial Products Pvt. Ltd., Ambala Cantt., Haryana, India) in a laboratory of the National Gene Bank of the Nepal Agricultural Research Council (NARC), Khumaltar, Lalitpur, Nepal. Solutions of various water potentials (0, -0.149, -0.51, -1.09, -1.88, -2.89, -4.12, and -5.56 MPa) were prepared using polyethylene glycol (PEG 6000; Qualigens fine chemicals Besant Road, Mumbai, India). The PEG solutions were prepared according to a published protocol [13] and the solutions were calibrated according to the protocols used by Shrestha et al. [14]. Twenty-five seeds of *A*. *houstonianum* were placed on Petri dishes lined with Whatman No. 1 filter paper. Although the upper limits of the water potentials selected are more negative than those for other studies that assessed water potential levels of up to -1.0 MPa on some Asteraceae species [9, 12], the treatments were selected to explore drought tolerance extremes as some species, e.g. *Echinochloa colona* seeds germinated up to -5.5 MPa [14]. Ten ml of the different water potential solutions were added to each Petri dish with a pipette. The Petri dishes were then sealed with parafilm and placed in the seed germination chamber set at a constant temperature of 20 ± 2°C. Light intensity inside the chamber was measured with a Solar Power Meter (Amprobe Solar-100, Everett, WA, USA). The light intensity was measured at five locations in the chamber and the average was 1.4 W m^-2^. The experimental setup was a completely randomized design, with each water potential treatment replicated eight times, and the experiment was repeated. The first and second round of the experiments were conducted in March/April 2018 and June/July 2018, respectively.

Seed germination was evaluated every day until germination ceased. A seed was considered to be germinated when a 1 mm or more radicle emerged from the seed. Once a seed had germinated, it was counted and removed from the Petri dish using tweezers. Germination usually ceased by the fourth day. Any seed that failed to germinate in each treatment was collected carefully with a tweezer, washed in distilled water, transferred to a Petri dish lined with a filter paper, 10 ml of distilled water was added, and the Petri dish was placed back into the chamber. This process provided an estimate of the viability of the seeds in each treatment when they failed to germinate. The cumulative numbers of germinated seeds were then expressed as percentages of the total number of viable seeds.

### Effect of buffer solution pH on seed germination

Experiments were conducted in 2018 in the same seed germination chamber described above. Ten ml of distilled water (pH 7) was used to prepare solutions of different pH level buffered solutions. For the acidic solutions (pH 4, 5, and 6), 1N hydrochloric acid (HCl) was used and for the alkaline (pH 8, 9) 1 N Sodium hydroxide (NaOH) was used. The pH levels were confirmed with a microprocessor pH meter (Hanna Instruments, Model pH213, Carrollton, TX, USA). Petri dishes were lined with Whatman No. 1 filter paper and then 10 ml of the buffered solutions with various pH levels were added to each petri dish (according to the assigned treatment) with a pipette using the procedures described by Chachalis and Reddy [15]. Twenty-five seeds of *A*. *houstonianum* were then placed in each Petri dish, were sealed with parafilm, and placed in the germination chamber set at temperature and light levels described earlier. Seed germination evaluation protocols were similar to that described earlier. Germination usually ceased by the sixth day. The experiment was arranged as a completely randomized designed with eight replications per treatment and was conducted twice. The first and second round of the experiments were conducted in April/May 2018 and June/July 2018, respectively.

### Effect of light on seed germination

Experiments were conducted in 2018 in the seed germination chamber described above to evaluate the effect of complete darkness and ambient light (1.4 W m^-2^) in the chamber on seed germination. Twenty-five seeds of *A*. *houstonianum* were placed in Petri dishes lined with Whatman No. 1 filter paper. Ten ml distilled water were added to each Petri dish with a pipette. The complete darkness treatment was created by wrapping individual Petri dishes with aluminum foil whereas the light treatment Petri dishes were left unwrapped. The Petri dishes were then sealed with parafilm and placed in the seed germination chamber with the temperature and light set as described above. Similar procedures, as described earlier, were used to evaluate germination. However, evaluation was continued up to ten days to assess the germination of the seeds under the dark treatment. After the tenth day the aluminum foil was removed, and the Petri dishes were placed back in the chamber to assess seed viability and most of the seeds germinated within four days. The experiment was arranged as a completely randomized design with eight replications and was conducted twice. The first and second round of the experiments were conducted in April/May 2018 and June/July 2018, respectively.

### Effect of seed burial depth on seedling emergence

The experiment was conducted inside the same laboratory at normal room temperature (approximately 25° C). Each experimental unit was a 15 cm tall plastic pot, filled with soil (sandy loam) collected locally from the NARC experimental farm. The soil was autoclaved and passed through a 3-mm sieve. Equal amount of some pebbles was added to the base of all the pots to allow aeration. The pots were marked at the surface using a black permanent marker, this indicated a depth of 0 mm. Then 2, 5, 10, 15, and 20 mm depths were also marked on each pot with the help of a measuring ruler. Twenty seeds were placed on the soil surface in the 0 mm depth treatment pots. For the other treatments (2, 5, 10, 15, and 20 mm depths), soil was first filled to the corresponding depth, twenty seeds were then placed at the designated depth, and then soil was added to the surface of the pot; thus, burying the seeds at each targeted depths. Pots were sprinkle irrigated with tap water (pH of approximately 7.0) initially and later sub irrigated by placing them in trays filled with water. The experiment was terminated in three weeks when no further emergence was observed. The experiment was arranged as a randomized complete block design with each treatment replicated eight times and the study was repeated. The first and second round of the experiments were conducted in May/June 2018 and July/August 2018, respectively.

### Estimation of seed dormancy requirement

Seeds of *A*. *houstonianum* for this objective of the study were collected from the location described earlier. The seeds were tested for germination on, a) the day they were harvested, b) one week after they were harvested, c) two weeks after they were harvested, d) three weeks after they were harvested, and e) four weeks after they harvested. The seeds that were not tested for germination the day that they were collected were stored under room conditions as described earlier, till their designated day of treatment. Twenty-five seeds were placed in Petri dishes lined with Whatman No. 1 filter paper. Ten ml distilled water was added to each Petri dish with a pipette. The Petri dishes were then sealed with parafilm and placed in the germination chamber with the settings described earlier. Seeds were evaluated for germination every day using the protocols described earlier. The experiment was arranged as a completely randomized design with eight replications and conducted twice. Separate seeds were harvested from the plants in the same location and used in the second round of the study. The first round of the experiment was initiated on July 4, 2018 and the second round on July 22, 2018 respectively.

### Data analysis

For each experiment, the imposed treatments (water potential, pH, light, burial depth, and dormancy period) were considered as fixed effects, while replications and experimental runs were considered as random factors. All possible interactions between the fixed and the random effects were also tested. In all cases, data were tested to verify if the assumptions of ANOVA were met using the Shapiro-Wilk’s test for normality and Levene’s test for homogeneity of variance at a 0.05 level of significance. Data that failed to meet the assumptions of normality and were transformed using log10 procedure prior to analysis (data for the pH experiment did not need transformation). Data were analyzed using the software SAS v. 9.4 (SAS Institute, Cary, NC, USA) and means were separated using Fisher’s Least Significant Difference test whenever the ANOVA indicated significance. Since there was no treatment by experimental run for any of the experiments, data for the two runs were combined for each experiment. Nontransformed means with Fisher’s Least Significant Difference (LSD) separations based on transformed data are reported for ease in presentation. Data were further analyzed for the water potential and the seed dormancy experiment using nonlinear regression models and fitted with a sigmoidal three-parameter model (Sigma Plot v. 12.3), which took the following form:

$$y=a/(1+exp(-(x-x_{0})/b))$$

where *y* is the response variable; *a* is the upper asymptote (maximum); *x* is the fixed variable; *x*_*0*_ is the water potential or seed burial depth resulting in 50% value of *y*; and *b* is the slope of the curve at *x*_*0*_. For the seed dormancy study, a linear regression was fit to the germination data as a function of time of planting of the seeds after harvesting.

## Results and discussion

### Effect of moisture stress on seed germination

**Fig 1:**
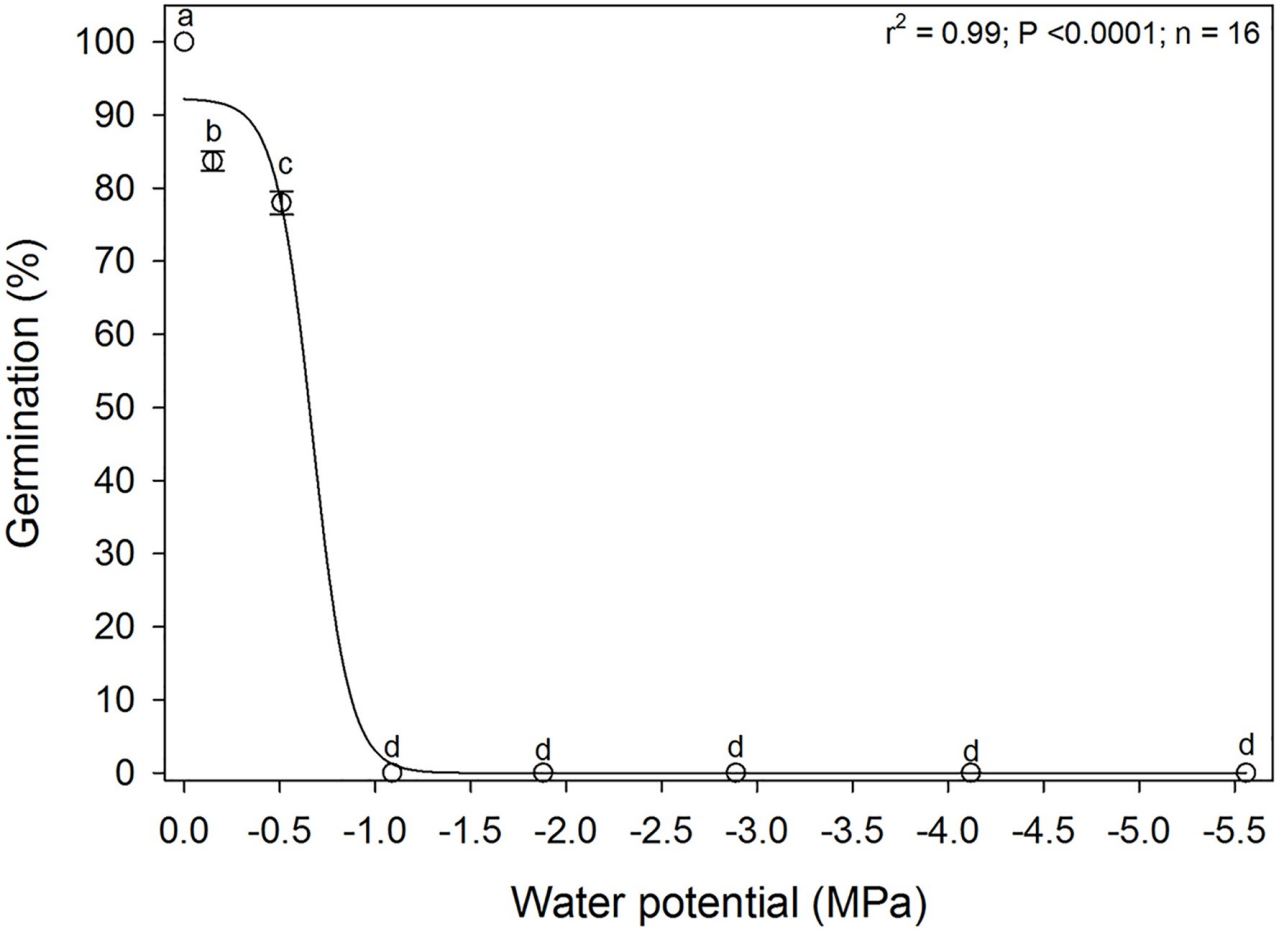
Effect of water potential on germination of *A*. *houstonianum* seeds incubated in a seed germination chamber at 20° C for 29 days. The regression line represents a three-parameter sigmoid model fitted to the data. Means with the same letters are not significantly difference according to the Fisher’s Protected LSD test at α = 0.05.

Germination of *A*. *houstonianum* seeds was significantly influenced by more negative levels of water potential (moisture stress) as induced by the different concentrations of PEG (Fig 1). Seed germination in the distilled water treatment (0 MPa) was considered 100% and germination under the other water potential treatments were expressed as relative to the 0 MPa treatment. Seed germination was significantly lower (approximately 84%) in the -0.149 MPa treatment compared to the control with a further reduction in germination at -0.51 MPa (approximately 78%), while none of the seeds germinated at the more negative water potential levels. From the non-linear regression it was estimated that the water potential level that reduced seed germination by 50% was approximated from the regression as -0.67 MPa. When the seeds that failed to germinate in the various treatments were washed with distilled water and placed back in the germination chamber in petri dishes with new filter paper, approximately 80% of the seeds exposed to various water potentials germinated, indicating that the seeds were still viable. These results indicated that *A*. *houstonianum* was only moderately tolerant to moistures stress at germination.

Several studies have assessed weed seed germination using PEG 6000 to impose drought stress [14, 16] including in Asteraceae species [11, 17]. It has been suggested that PEG reduces the water potential of osmotic solutions and thus can be used for drought stress studies [13]. A study reported that, although PEG solutions can be slightly unstable, they have no biological consequences in seed germination studies and induced drought stress by PEG showed similar values to that observed in fields [18]. In our study, there was no germination of *A*. *houstonianum* seeds in water potential levels more negative than -1.0 thus suggesting that this species was moderately tolerant to moisture stress and may not germinate in drier environments. Probably, the lack of moisture stress during germination is one of the reasons that this species is more prevalent in tropical and subtropical areas with more precipitation than in drier regions of the world or regions with irrigation. Similar results have been reported for other Asteraceae species. For example, germination of seven different species belonging to the Asteraceae species reported almost no germination beyond -0.5 MPa [17]. In another *Ageratum* species, *A*. *conyzoides*, it was observed that only about 20% of the seeds germinated at -1 MPa [9]. This is very similar to the findings of our study showing that this species is not as drought tolerant as *E*. *colona* during germination [14].

### Effect of pH levels on seed germination

**Fig 2:**
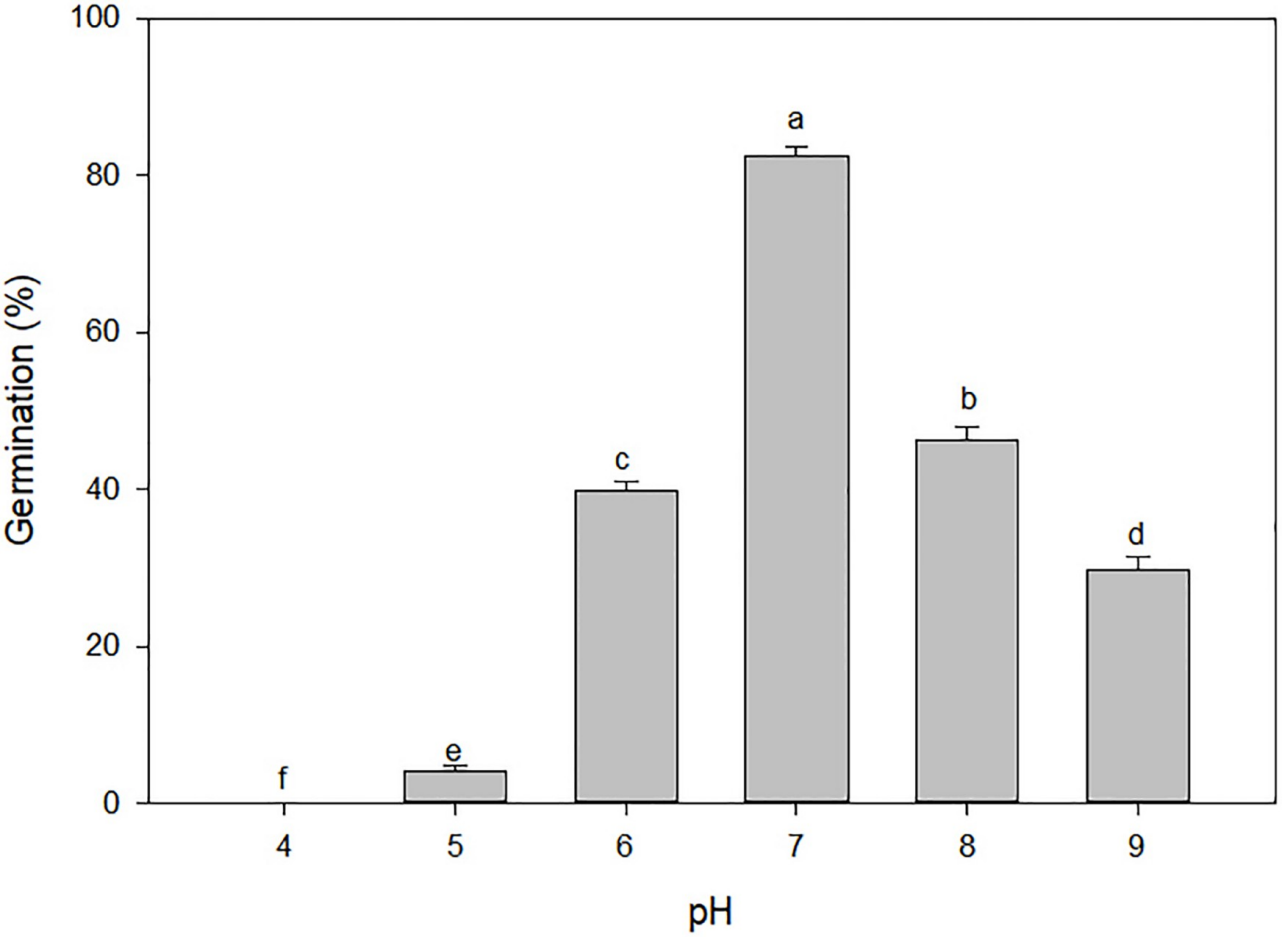
Effect of different pH levels on germination of *A*. *houstonianum* seeds incubated in a seed germination chamber at 20° C for 29 days. Means with the same letters are not significantly difference according to the Fisher’s Protected LSD test at α = 0.05.

The pH level of the solution media had a significant effect on the germination of *A*. *houstonianum* seeds. Germination was greatest (approximately 83%) at a pH level of 7 and declined at higher or lower pH treatments (Fig 2). Unlike in the moisture stress study, actual data on germination is presented and not normalized to the pH 7 data. Compared to the pH 7 treatment, germination was reduced by 45% and 52% at the pH treatments of 8 and 6, respectively. Germination was completely inhibited in the lowest pH treatment (pH 4). However, about 30% of the seeds germinated at the highest pH level tested (pH 9). When the seeds that failed to germinate in the various pH treatments were washed with distilled water and placed back in the germination chamber in petri dishes with new filter paper, approximately 80% of the seeds exposed to pH levels of 6, 7, 8, and 9 germinated indicating that the seeds were still viable. However, none of the seeds exposed to the pH levels of 4 and 5 germinated and they were infested with fungal growth. These results indicated that *A*. *houstonianum* seeds preferred neutral to alkaline pH conditions than acidic conditions for germination.

Soil pH is an important environmental factor affecting seed germination and various studies have been conducted to assess the effect of pH on germination of weed seeds [e.g. 15] including some Asteraceae species [12, 19]. In our study, germination of *A*. *houstonianum* seeds occurred between a pH range of 6 and 9 with maximum germination at pH 7. This suggested that neutral-to-alkaline soils were more suited for its germination than acidic soils. Similar results in *A*. *houstonianum* with comparatively more germination occurring at higher than lower pH levels and the highest amount of germination at pH 7 have been reported [19]. Furthermore, similar results have also been reported in other invasive Asteraceae species where optimum pH level for germination was 7 with a tendency for greater germination under alkaline than acidic conditions. Examples include *Conyza canadensis* [12] and *Asclepias syriaca* L. [20].

### Effect of light on seed germination

**Fig 3:**
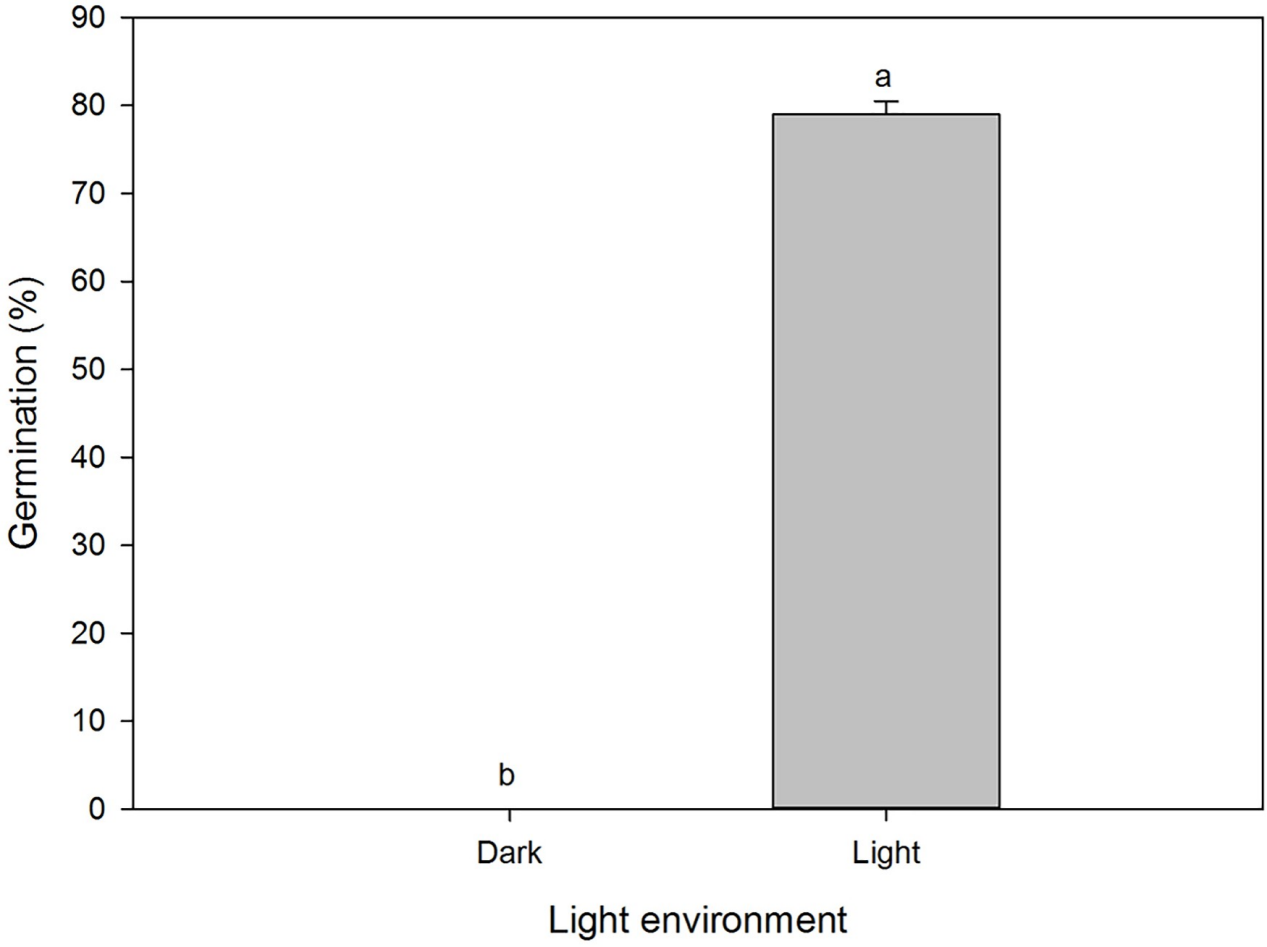
Effect of light on germination of seeds of *A*. *houstonianum* incubated in a seed germination chamber at 20° C for 29 days. Means with the same letters are not significantly difference according to the Fisher’s Protected LSD test at α = 0.05.

Germination of *A*. *houstonianum* seeds under the dark and light conditions were significantly different (Fig 3). Approximately, 80% of the seeds germinated in the petri dishes that were exposed to the light settings (1.4 W m^-2^) in the growth chamber, whereas none of the seeds in the petri dishes that were wrapped in aluminum foil (dark conditions) germinated. However, when the aluminum foils were removed from the petri dishes and placed back in the germination chamber after 29 days, most of the seeds (approximately 80%) of the seeds germinated indicating that the seeds were still viable. These results suggested that light was a requirement for seed germination in this species. However, it is not known what level of light intensity was required for germination as the results of the study is limited to the light setting in the germination chamber.

Light is an absolute requirement for germination of several weed species [20] including Asteraceae species [21], especially those species that have small seeds [22]. It was reported that seed germination was greater in some Asteraceae species with smaller than larger seeds under light than in dark conditions [21]. The average size of *A*. *houstonianum* seeds used in this study was 1.5 mm with a 2.5 to 3 mm long pappus attached to it. Therefore, the seeds of this species fall in the small category. Similarly, it was reported that light was a requirement for germination of ten Asteraceae species which had individual seed weights less than 0.5 mg [23]. Greater germination under light than dark conditions were recorded in *C*. *canadensis* [12]. In our study, seeds of *A*. *houstonianum* germinated only in the presence of light whereas, no germination was recorded in the absence of light. This suggested that this species has photoblastic seeds as they demonstrated positive photosensitivity and germination of this species is a photochrome-mediated response, which is an attribute commonly observed among weeds [24]. It has been reported that seeds of *A*. *conyzoides* were photoblastic and germination was phytochrome mediated [9]. Similar results have been reported in some other *Conyza* spp. [25, 26]. However, it is not known what the minimum light intensity level was for germination of *A*. *houstonianum*. Since the light level in the germination chamber was quite low (approximately 1.4 W/m^2^), it can be suggested that seed germination in this species may not require very high light intensities, but some level of light is required. Yuan and Wen [9], as mentioned above, conducted their study on *A*. *conyzoides* with a 12 h photoperiod of 25 μmol m^-2^ s^-1^.

### Effect of seed burial depth on seedling emergence

**Fig 4:**
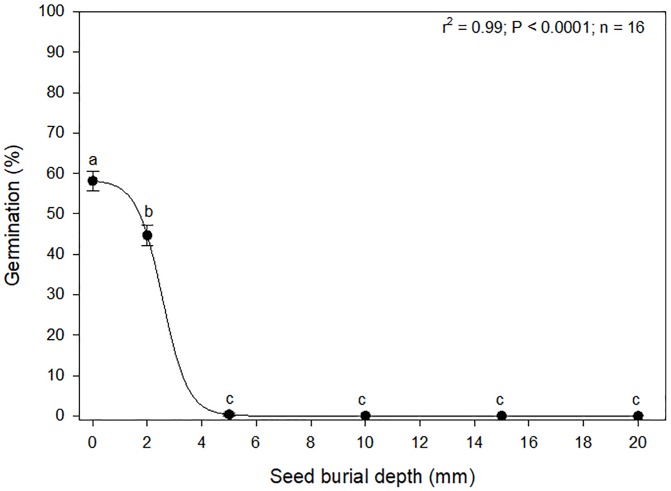
Effect of seed burial at various soil depths on seedling emergence of *A*. *houstonianum*. Means with the same letters are not significantly difference according to the Fisher’s Protected LSD test at α = 0.05.

Seed burial depth had a significant effect on the emergence of *A*. *houstonianum* seedlings. Again, unlike in the moisture stress study, actual data on germination is presented and not normalized to any of the burial depths. Seedling emergence was greatest (50–55%) for seeds placed on the soil surface, followed by seeds buried at 2 mm (40–45%) (Fig 4). No seedlings emerged from the seeds that were buried more than 2mm deep. From the non-linear regression, it was estimated that soil depth for 50% inhibition (compared to that at the soil surface) of seedling emergence was approximately 2.5 mm. These results suggested that *A*. *houstonianum* was a primarily a surface-emerging species and seedling emergence of seed buried deeper than 2 mm depths would be significantly reduced or even completely inhibited. However, it is now known if the seeds buried at the deeper depths germinated but failed to emerge.

This study showed that *A*. *houstonianum* was species with seeds adapted to primarily surface-germination and emergence. No seeds that were buried more than 2 mm deep in the soil emerged. Such emergence patterns have been reported in several Asteraceae weed species such as *C*. *canadensis* [12]. This is probably an adaptation strategy of photoblastic species. As discussed earlier, *A*. *houstonianum* is photoblastic and needs light for germination. It can also be implied that lack of emergence from seeds buried deeper in the soil profile may be primarily due to absence of light. The ecological significance attributed to the light response in these species is that light acts as a soil depth “indicator,” allowing greater germination of surface seeds than seeds buried in soil [21]. However, it was not determined if the seeds buried deeper than 2 mm failed to germinate or if they did germinate but failed to emerge. It is speculated that it may be the former case and the seeds remain photo-dormant because studies have reported that seeds buried more than 0.2 cm below the soil surface usually receive less than 1% of the incident light [27, 28]. Our findings are similar to reports for several other weed species that lack a light trigger for germination [29]. Therefore, the germination and emergence of this species may be reduced if they are buried more than 2 mm in the soil and this could be a possible option to manage the rapid spread of this species. The longevity of the seeds in the soil seedbank is not known; however, a study reported that seeds of A. *conyzoides* start losing their vigor within a year [30]. Survival of the seeds in the soil seedbank are influenced by various factors such as dormancy state, germination cues, seed size, and burial depths [31, 32]. It has been reported that seeds of *C*. *bonariensis*, which has similar size, germination, and emergence characteristics as *A*. *houstonianum* had a longevity of about three years in the soil seedbank [33]. Therefore, the seeds of *A*. *houstonianum* may also have similar longevity in the soil seedbank.

### Seed dormancy period required for germination

**Fig 5:**
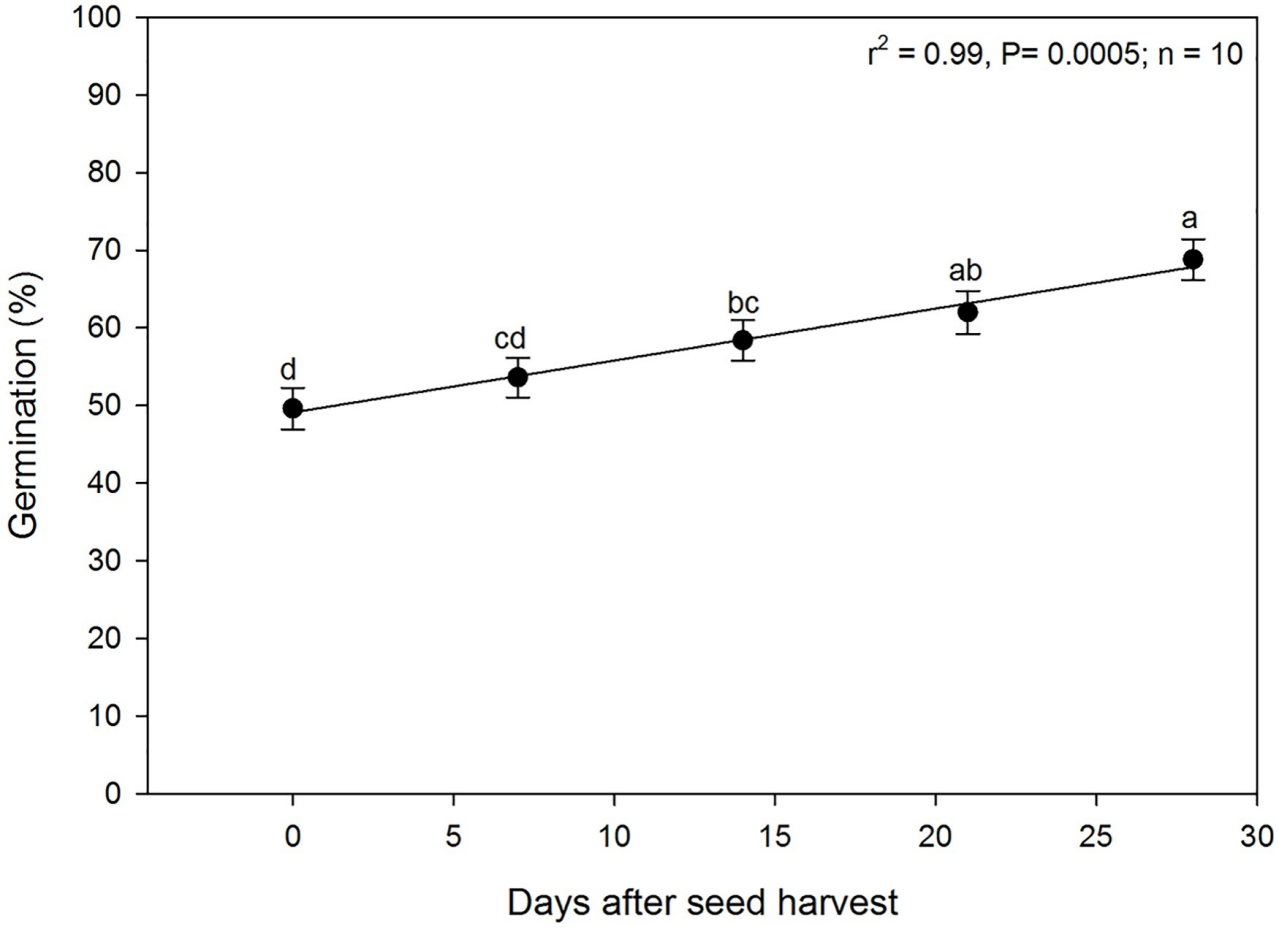
Germination of *A*. *houstonianum* seeds at various days after seed harvest. Means with the same letters are not significantly difference according to the Fisher’s Protected LSD test at α = 0.05.

Germination of the seeds was significantly affected by the number of days they underwent dormancy (Fig 5). Germination increased linearly with the number of days the seeds were kept dormant (i.e. stored under lab conditions). Germination was greatest (approximately 65 to 70%) when the seeds were stored for four weeks and then tested. It is not known if the germination would have been higher if the seeds were stored longer as the treatments did not exceed four weeks of storage. However, the germination of the seeds, in general, in all the studies did not exceed 85%. Almost 50% of the seeds that were planted on the day they were collected germinated. This suggested that seeds *A*. *houstonianum* had a very short dormancy period and could germinate the day they fell of the plant, provided that they were exposed to suitable environmental conditions such as moisture, temperature, and light.

This study showed that seeds of *A*. *houstonianum* had the ability to germinate the day they fall of the mother plants. Although percent seed germination increased linearly with increase in storage period, this species does not seem to have a very long dormancy period because most of the seeds germinated within a month. Many seeds undergo a period of dormancy after dispersal–such a period could be a few days to many decades or longer [34]. Furthermore, seeds can also remain viable for extended period. For example, seeds of *Malva neglecta* can remain viable for over 100 years [35]. Long dormancy, or delayed germination, can be disadvantageous when fast colonization is required [36]. An absence of dormancy can also be a disadvantage due to the reduction of dispersal opportunities [34]. It was reported that dormancy is an advantage in environments where adult populations are destroyed by disturbance [36]. Lack of dormancy periods in *A*. *houstonianum* suggests that this species can spread rapidly in an area as the seeds can germinate as soon as they fall from the mother plants provided that adequate soil moisture and light is present, and that the seeds are not buried more than 2 mm deep in the soil or covered by debris, mulch, or litter. However, the longevity of the seeds in the seedbank needs to be determined.

## Conclusions

This study indicated that seeds of *A*. *houstonianum* were not very drought tolerant during germination and may be better suited to rainy climates or irrigated areas. Greater germination would occur under neutral to alkaline soil conditions than under acidic soil conditions. Also, the seeds were positively photoblastic and would need some amount of light to germinate. *A*. *houstonianum* species is primarily a surface-emerging species and seeds buried more than 2 mm deep in the soil would most likely not emerge. Furthermore, this study showed that seeds of this species had no dormancy period and most of the seeds could germinate immediately after falling from the mother plant, provided the other environmental factors as discussed above were suitable. Thus, it is predicted that this weed has the ability to invade most of the areas of Nepal under a range of moisture and soil conditions as long as the seeds remain on the soil surface and are not buried or covered by litter or other substances. In agricultural lands, soil disturbance in the form of light tillage or cultivation would probably reduce its germination ability. Such information could be used in developing management strategies for this weed species, reduce invasions, and to develop mathematical models for population dynamics to predict future invasions.

## Supporting information

S1 Fig (XLSX)

S2 Fig (XLSX)

S3 Fig (XLSX)

S4 Fig (XLSX)

S5 Fig (XLSX)
